# Environmental and Social Change Drive the Explosive Emergence of Zika Virus in the Americas

**DOI:** 10.1371/journal.pntd.0005135

**Published:** 2017-02-09

**Authors:** Sofia Ali, Olivia Gugliemini, Serena Harber, Alexandra Harrison, Lauren Houle, Javarcia Ivory, Sierra Kersten, Rebia Khan, Jenny Kim, Chris LeBoa, Emery Nez-Whitfield, Jamieson O’Marr, Emma Rothenberg, R. Max Segnitz, Stephanie Sila, Anna Verwillow, Miranda Vogt, Adrienne Yang, Erin A. Mordecai

**Affiliations:** Department of Biology, Stanford University, Stanford, California, United States of America; North Carolina State University, UNITED STATES

## Abstract

Since Zika virus (ZIKV) was detected in Brazil in 2015, it has spread explosively across the Americas and has been linked to increased incidence of microcephaly and Guillain-Barré syndrome (GBS). In one year, it has infected over 500,000 people (suspected and confirmed cases) in 40 countries and territories in the Americas. Along with recent epidemics of dengue (DENV) and chikungunya virus (CHIKV), which are also transmitted by *Aedes aegypti* and *Ae*. *albopictus* mosquitoes, the emergence of ZIKV suggests an ongoing intensification of environmental and social factors that have given rise to a new regime of arbovirus transmission. Here, we review hypotheses and preliminary evidence for the environmental and social changes that have fueled the ZIKV epidemic. Potential drivers include climate variation, land use change, poverty, and human movement. Beyond the direct impact of microcephaly and GBS, the ZIKV epidemic will likely have social ramifications for women’s health and economic consequences for tourism and beyond.

## Introduction

**Fig 1:**
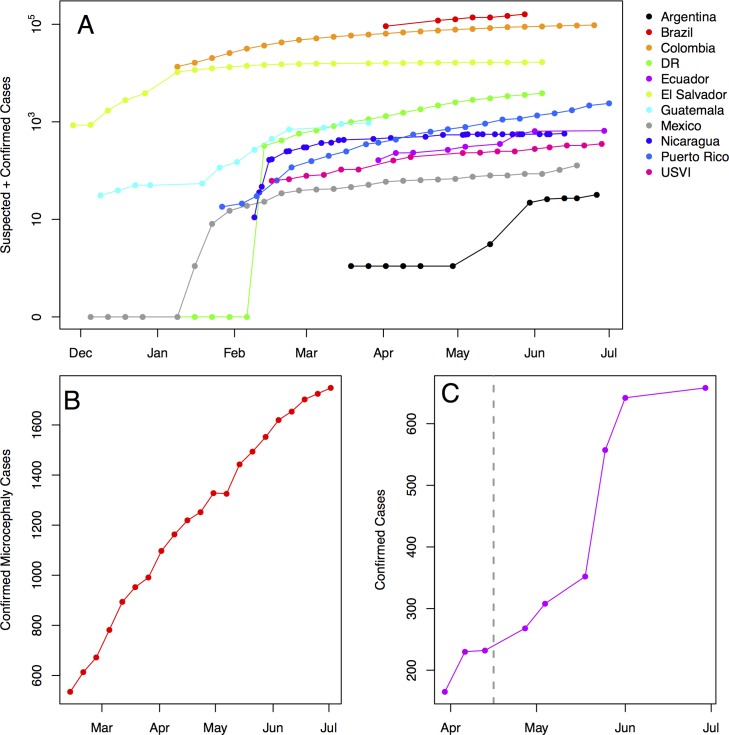
ZIKV epidemic dynamics in 2015–2016 across the Americas, in Brazil, and in an Ecuadorian province struck by an earthquake. (A) Weekly cumulative suspected and confirmed ZIKV cases (see Glossary for case definitions) across countries in the Americas, on a log scale, colored by the total size of the epidemic. (B) Weekly cumulative confirmed ZIKV-linked microcephaly cases (see Glossary for case definitions) in Brazil. (C) Weekly cumulative confirmed autochthonous cases in Manabi province, Ecuador, where a magnitude 7.8 earthquake struck on April 16, 2016 (indicated by the dashed line). Data are from weekly epidemiological reports from the Pan American Health Organization (PAHO), as compiled by the Centers for Disease Control and Prevention Epidemic Prediction Initiative (CDC EPI; https://github.com/cdcepi/zika; accessed 15 July 2016).

**Fig 2:**
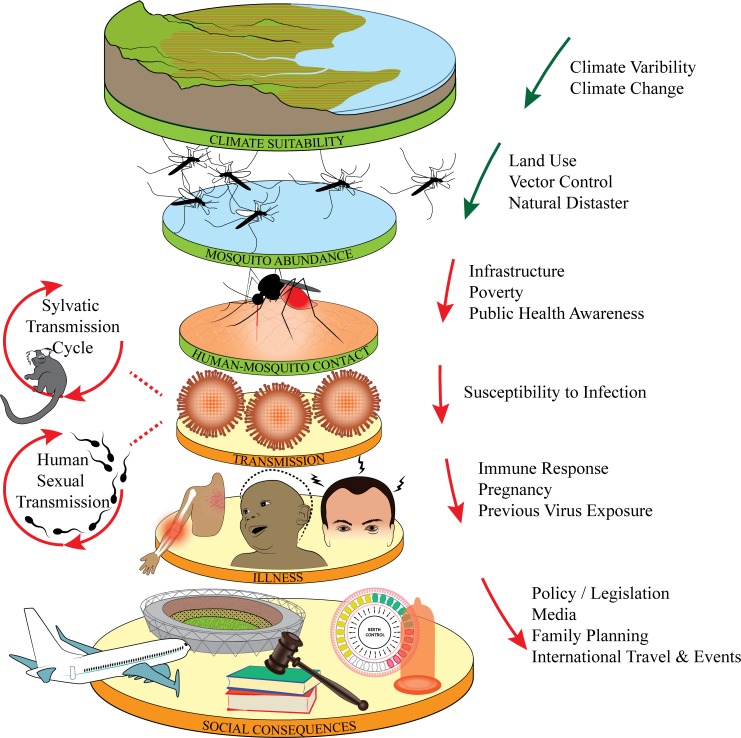
Hierarchy of factors that influence ZIKV transmission, illness, and social consequences. Climate suitability, mosquito abundance, and human–mosquito contact partly determine rates of ZIKV transmission, which causes illness in some cases. Social consequences depend on both actual and perceived risks of illness. Arrows indicate environmental (green) and social (red) changes hypothesized to contribute to the shifting ecology of vector transmission in the Americas. Figure inspired by Plowright et al. [22].

Over the past four decades, the global emergence and resurgence of arboviruses has become a burgeoning public health crisis. Dengue virus (DENV; family *Flaviviridae*, genus *flavivirus*), which began reappearing globally in the late 20th century, more than quadrupled in reported incidence from the 1980s–2000s and now causes an estimated 96 million symptomatic cases yearly [1–3]. In 2013, chikungunya virus (CHIKV; family *Togaviridae*, genus *alphavirus*) emerged for the first time in the Americas in St. Martin, causing 1.8 million suspected and confirmed cases in the region [4]. In 2015, Brazil confirmed the first case of locally acquired Zika virus (ZIKV; family *Flaviviridae*, genus *flavivirus*) infection in the Americas [5]. The ZIKV epidemic has since caused over 500,000 suspected and confirmed cases in 40 countries and territories in the Americas [6,7] (Fig 1). Many ZIKV cases have likely gone unreported due to the initial low reporting, high proportion of asymptomatic cases (>80%) [8], and diagnostic challenges (including overlapping symptoms and immunological cross-reactivity with DENV, which is also a flavivirus). Since ZIKV and DENV antibodies are structurally similar, antibody cross-reactivity between ZIKV and DENV may even enhance ZIKV infection (Fig 2) [9]. Mutations in the ZIKV genome may also contribute to ZIKV infection [10]. As of now, mutations have been identified but none have been linked to the rapid spread of ZIKV or the severity of its complications [10], including microcephaly and Guillain-Barré syndrome (GBS) [6,11]. With strikingly similar paths of arrival and spread through the Americas, the ongoing DENV, CHIKV, and ZIKV epidemics represent a dangerous new pattern of arbovirus emergence and resurgence that demands renewed focus on the global ecology of vector transmission.

These (re)emerging arboviruses are diseases of global movement and change. They share a primary mosquito vector (*Aedes aegypti*) that lives alongside human settlements, oviposits in household water containers, and feeds preferentially on humans [12–14]. During the 16th and 17th centuries, *Ae*. *aegypti* and yellow fever virus (YFV; family *Flaviviridae*, genus *flavivirus*), also transmitted by this vector, spread from West Africa to the Americas with the slave trade [15]. DENV followed, expanding worldwide along shipping routes in the 18th and 19th centuries [16] and circulating throughout Southeast Asia after World War II. In Latin America, DENV declined with aggressive vector control in the 1950s–1970s but has since resurged, in part as a result of decreased prioritization of vector control efforts [1] and increased mosquito pesticide resistance [17]. CHIKV and ZIKV were first isolated and described in Africa in the 1940s–1950s [18], though extensive continental spread of both viruses likely occurred prior to their discovery [19–21]. Like DENV, they spread into Southeast Asia in the 1960s–1970s, then began causing major outbreaks on islands several decades later before sweeping through the Americas starting in 2013 and 2015, respectively [18]. This shared history of widespread circulation in Africa, emergence in Southeast Asia, and recent explosive epidemics in Pacific and Indian Ocean Islands and the Americas suggests a changing global ecology of transmission by the shared *Aedes* spp. vectors, with global change intensifying transmission (Fig 2).

Global change includes a suite of anthropogenically-driven factors that intensify with population growth and are changing in concert, including climate, land use, urbanization, social and political policies, poverty, and human movement. These factors interact to influence mosquito distribution and abundance, human contact, and association with arboviruses (Fig 2). How have intensifying environmental and social factors shaped the global ecology of vector transmission to allow ZIKV to emerge in the Americas? Here, we evaluate the evidence for a range of human-induced environmental and social changes that may have led to ZIKV emergence and the social consequences that may arise from the ZIKV epidemic.

### Environmental drivers of ZIKV emergence

The establishment of autochthonous (local) ZIKV transmission usually occurs in an urban transmission cycle [23]. *Ae*. *aegypti* mosquitoes have high vector competence [13,24], making them highly effective vectors for ZIKV and other arboviruses. ZIKV can also infect *Ae*. *albopictus*—a secondary vector of CHIKV, DENV, and likely ZIKV [25,26]—and many other *Aedes* spp. mosquitoes [14]. Once introduced, ZIKV can begin an urban cycle anywhere with sufficient mosquito and human populations or a sylvatic transmission cycle if it is present in wildlife-feeding mosquito populations. This intimate connection between ZIKV and the mosquito life cycle makes transmission highly sensitive to the environmental conditions that affect *Ae*. *aegypti* survival and abundance, including temperature and availability of breeding sites.

### Climate variation and change

Weather and climate shape mosquito geographic distribution, population abundance, lifespan, and transmission potential. Based on the nonlinear effects of temperature on virus incubation rate and rates of fecundity, development, survival, and biting for *Ae*. *aegypti* and *Ae*. *albopictus* [27], recent work suggests that ZIKV transmission can occur between 18°C and 34°C, with a peak at 29°C [27]. In the Americas, ZIKV has emerged primarily in tropical and subtropical zones where summer temperatures are already highly suitable for *Ae*. *aegypti*, supporting the role of climate suitability in driving ZIKV transmission (Fig 2) [28]. However, climate variation and/or change may have increased year-round temperatures to the optimal levels for ZIKV transmission, lengthening the transmission season in tropical and subtropical regions. For example, in 2015, Brazil’s winter and spring were among the warmest and driest on record due to a strong El Niño and possibly climate change [29]. Warmer winter temperatures, which also promote human water storage practices that can increase the number of *Ae*. *aegypti* breeding sites [30], may have placed much of Brazil into transmission-permissive conditions year-round in 2015, potentially supporting ZIKV spread during a typically low-transmission season. However, this connection remains poorly supported to date.

Climate variation and/or change may also be a concern in temperate regions where year-round temperatures are not typically suited to *Ae*. *aegypti*. There, warmer winter temperatures can increase *Ae*. *aegypti* overwinter egg survival, which is restricted to the 10°C winter average temperature isotherm, potentially expanding *Ae*. *aegypti’s* range to higher latitudes and altitudes [31,32]. While warmer winter temperatures may expand the geographic range of *Ae*. *aegypti* and its arboviruses, warmer spring, summer, and fall temperatures may extend the length of the transmission season in temperate areas as well, especially in regions where *Ae*. *aegypti* or *Ae*. *albopictus* are locally established, such as Italy and the northeastern United States [27,33,34]. As a result, climate change and/or variation may influence the geography of vector transmission and intensify the threat of ZIKV in temperate regions.

### Rural–Urban gradient, land use change, and deforestation

**Fig 3:**
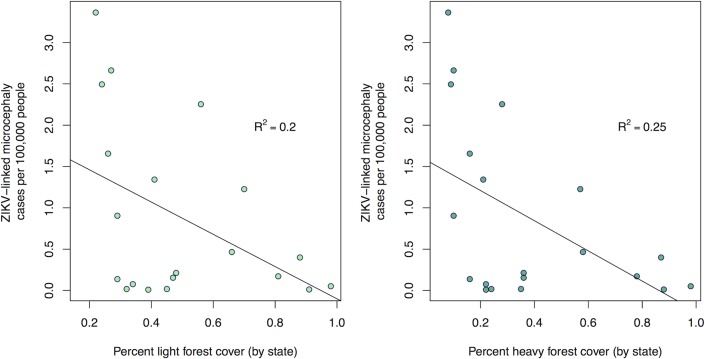
Confirmed ZIKV-linked microcephaly cases in Brazilian states versus percent light and dense forest cover. The number of confirmed ZIKV-linked microcephaly cases per 100,000 people (as of July 15, 2016) in Brazilian states with (A) light forest cover (r = -0.27) and (B) dense forest cover (r = -0.35) is shown for states that reported one or more cases. For each state, each hectare with >10% and >50% forest cover is added to the forest cover total for light and dense forest cover calculations, respectively. The *x*-axis shows forest cover as a percentage of total land area, based on light (A) and dense (B) forest calculations. Microcephaly data are from PAHO case reports, as compiled by the CDC EPI. Population sizes by state were retrieved from the 2014 Brazilian Institute of Geography and Statistics (IBGE) report (ftp://ftp.ibge.gov.br/Estimativas_de_Populacao/Estimativas_2014/estimativa_dou_2014.pdf; accessed 7 July 2016). Forest cover data are from http://rainforests.mongabay.com/20brazil.htm (accessed July 7, 2016).

The rural–urban gradient clearly influences ZIKV vectors, *Ae*. *aegypti* and *Ae*. *albopictus* [35,36]. We examined the relationship between the per capita rate of confirmed ZIKV-linked microcephaly cases, a proxy for total ZIKV cases, and the percentage of forest cover in each Brazilian state with one or more cases. We used confirmed ZIKV-linked microcephaly cases as a proxy for total cases because they are more likely to be reported than ZIKV cases in the general population (particularly asymptomatic cases), the data were available across states in Brazil, and they are expected to be strongly correlated with total ZIKV cases. We compared confirmed ZIKV-linked microcephaly cases to the percent of light forest cover (percent of hectares with >10% forest cover) and dense forest cover (percent of hectares with >50% forest cover). Both light and dense forest cover were negatively correlated with the prevalence of confirmed ZIKV-linked microcephaly cases (Pearson’s correlation coefficient r = -0.27 and Pearson’s correlation coefficient r = -0.35, respectively; Fig 3). Areas with less forest cover likely have higher human population densities, urbanization, suitability for *Ae*. *aegypti*, and ZIKV incidence, so human activities like deforestation and urbanization that decrease forest cover may increase the risk of ZIKV transmission.

### Animal reservoirs and the zoonotic transmission cycle

While *Ae*. *aegypti* is critical for human ZIKV epidemics, ZIKV also persists in a sylvatic transmission cycle that involves animal reservoirs [23]. The discovery of ZIKV in a sentinel rhesus monkey (a nonnative species) in Uganda [18], along with evidence of ZIKV antibodies in human blood samples collected in Africa in the 1950s–1960s [20], implies a sylvatic cycle. For ZIKV, as with other vector-borne zoonoses, urban and sylvatic cycles are likely maintained by different mosquito species, with occasional spillover by bridge vectors that feed on both animals and humans [23,37]. Human activity in areas where a sylvatic cycle is propagating can lead to human infection [37], which can then initiate an urban cycle with transmission via anthropophilic vectors like *Ae*. *aegypti*.

If ZIKV emerged from an animal reservoir into human populations, then significant contact between humans and infected animals may have preceded the human epidemic. We examined published seropositive rates of ZIKV in animal species in relation to their phylogenetic relatedness and physical proximity to humans. Despite living in close proximity to humans, all livestock species studied had low prevalence of ZIKV antibodies (Table 1). By contrast, some African primates, rodents, and birds—particularly those living in intact forest—had relatively high ZIKV seroprevalence, indicating an ongoing sylvatic cycle (Table 1). The highest risk of spillover to humans may be from coinhabiting primates, which are both phylogenetically and physically close to humans. Two such Brazilian primate species sampled between July and November 2015—capuchin monkeys and common marmosets—had high rates of ZIKV seroconversion (Table 1). Both are highly adaptable and thrive near industrialized cities [38,39]. While increased contact between wild animal reservoirs, sylvatic mosquitoes, and human populations as a result of land use change may have supported ZIKV emergence, the most important role of the sylvatic cycle going forward may be in maintaining a virus reservoir between human epidemics.

**Table 1:** Seropositive rates of ZIKV for selected animals in Uganda, Borneo, Pakistan, Kenya, and Brazil.

Host Species	Location	Habitat	Percentage of Population with Zika Virus Antibodies	
Wild Animal				
Colobus Monkey	Bwamba, Uganda	Intact Forest	45% (*n* = 11)	[40]
Redtail Monkey	Kisubi, Uganda	Intact Forest	19% (*n* = 21)	[40]
Redtail Monkey	Bwamba, Uganda	Intact Forest	19% (*n* = 21)	[40]
Mona Monkey	Bwamba, Uganda	Intact Forest	44% (*n* = 52)	[40]
Mangabey	Bwamba, Uganda	Intact Forest	50% (*n* = 2)	[40]
Orangutan	Borneo	Intact Forest	13% (*n* = 40)	[41]
Indian Desert Jird	Pakistan	Habitat Not Specified	6% (*n* = 33)	[42]
Indian Gerbil	Pakistan	Habitat Not Specified	2% (*n* = 47)	[42]
African Sacred Ibis	Kenya	Habitat Not Specified	4% (*n* = 49)	[43]
Cattle Egret	Kenya	Habitat Not Specified	3% (*n* = 37)	[43]
Ruffe	Kenya	Habitat Not Specified	50% (*n* = 2)	[43]
African Grass Rat	Kenya	Habitat Not Specified	4% (*n* = 1,446)	[43]
Kaiser's Rock Rat	Kenya	Habitat Not Specified	34% (*n* = 250)	[43]
Shrew	Kenya	Habitat Not Specified	3% (*n* = 63)	[43]
Livestock				
Sheep	Pakistan	Habitat Not Specified	2% (*n* = 46)	[42]
Goat	Pakistan	Habitat Not Specified	2% (*n* = 48)	[42]
Cattle	Kenya	Undeveloped Land	0.7% (*n* = 963)	[43]
Goat	Kenya	Adjacent to Irrigated Land	0.1% (*n* = 655)	[43]
Sheep	Kenya	Adjacent to Irrigated Land	0.7% (*n* = 283)	[43]
Cattle	Kenya	Adjacent to Irrigated Land	.5% (*n* = 1,361)	[43]
Coinhabiting Primates				
Orangutan	Borneo	Rehabilitation Center	3% (*n* = 31)	[41]
Capuchin Monkey	Brazil	Pets/Near Humans	33% (*n* = 9)	[44]
Common Marmoset	Brazil	Free-Ranging/Near Humans	27% (*n* = 15)	[44]

### Social drivers of ZIKV emergence

Social changes interact with environmental changes to promote ZIKV emergence and spread. For example, a number of social changes that expose people to a high density of infected mosquitoes emerge during and after natural disasters. In Ecuador, increased local ZIKV transmission occurred following the earthquakes that struck the Manabi province in April 2016 (Fig 1C), most likely by destroying infrastructure, contaminating drinking water (leading to makeshift water storage), and forcing people to live outdoors [45], all of which increase the rate of human contact with ZIKV-infected *Ae*. *aegypti* mosquitoes. Natural disasters can also interfere with the delivery of health care services, vector control, and education programs [46]. Social, political, and economic changes can also impact human exposure to ZIKV, particularly for the urban poor, who often live in areas with inadequate sanitation, infrastructure, and water access [47]. Shifting public sanitation policy, vector control efforts, and human movement can exacerbate existing discrepancies in these services. These social drivers have contributed to the shifting global ecology of vector transmission that has enabled ZIKV to emerge in the Americas by dangerously uniting human host, vector, and pathogen.

### Vector control

ZIKV transmission is closely connected to the abundance of *Ae*. *aegypti*, so changes in the efficacy of vector control may coincide with shifting prevalence of ZIKV and other *Ae*. *aegypti*-transmitted viruses. Historically, vector control has affected the global ecology of YFV and DENV transmission. In 1901, aggressive vector control reduced YFV cases more than 50-fold in a single year, from 1,400 cases in 1900 to 37 cases in 1901 [48], with similar results during construction of the Panama Canal [49]. Following World War II, the widespread availability of the pesticide DDT shifted public health responses to YFV from primarily emergency action to a large scale *Ae*. *aegypti* eradication program in Central and South America [50]. These efforts successfully eliminated *Ae*. *aegypti* and reduced outbreaks of YFV and DENV in large areas of the Americas from 1946 to 1970 [2,49]. A victim of their own success, vector control programs declined in the late 20th century [51], contributing to the dramatic resurgence of *Ae*. *aegypti* and DENV incidence [51,52]. Further, the unregulated use of insecticides to control DENV outbreaks has since allowed widespread resistance to develop in *Ae*. *aegypti*, exacerbating existing problems with modern vector control even amidst growing concerns about pesticide use [17]. Clearly, effective and coordinated vector control is necessary to suppress mosquito populations and, by extension, epidemics of arboviruses.

In 2002, Brazil instituted a National Plan for Dengue Control (PNCD) to address the recurring threat of DENV using source reduction, ultra-low volume space spraying of insecticides, and larvicide application [51]. While DENV incidence in Brazil initially dropped from 701,335 cases in 2002 to 72,552 cases in 2004, it quickly rebounded to 981,276 cases in 2010 [51], suggesting that transmission reductions from the ongoing PNCD were short-lived. Despite past success, classical vector control strategies, largely unchanged over the last 50 years, have not kept pace with the modern ecology of vector transmission. New self-limiting and self-sustaining approaches to vector control such as releasing genetically modified virus-refractory mosquitoes have been proposed [53] and have achieved some limited success [54], but they remain preliminary and hotly debated.

### Public sanitation policy, poverty, and recession

Public sanitation, including reliable access to clean piped water, garbage disposal, and sewage treatment, is one of the most essential public services that governments perform. In Brazil, the urban housing crisis, which is reinforced by large socioeconomic disparities, magnifies existing gaps in public sanitation services. In 2010, about 11.4 million people in Brazil lived in favelas (slums) on the outskirts of metropolitan areas [55]. Life in a favela implies life without infrastructure. Most slums lack effective sewage systems, access to potable water, and waste management [47], leading to increased water storage, especially in southeastern states where water storage in household containers accounts for more than 50% of mosquito breeding sites [12]. Changes in the quality and availability of public sanitation services may influence human exposure to ZIKV by increasing human–mosquito contact (Fig 2). Historically, public sanitation efforts in Brazil have routinely shifted between public and private funding, but sanitation policy is currently under government regulation [56]. Still, Brazil struggles to guarantee public sanitation to its poorest citizens [56], many of whom live in the northeastern states first and hardest hit by ZIKV [47,55].

**Fig 4:**
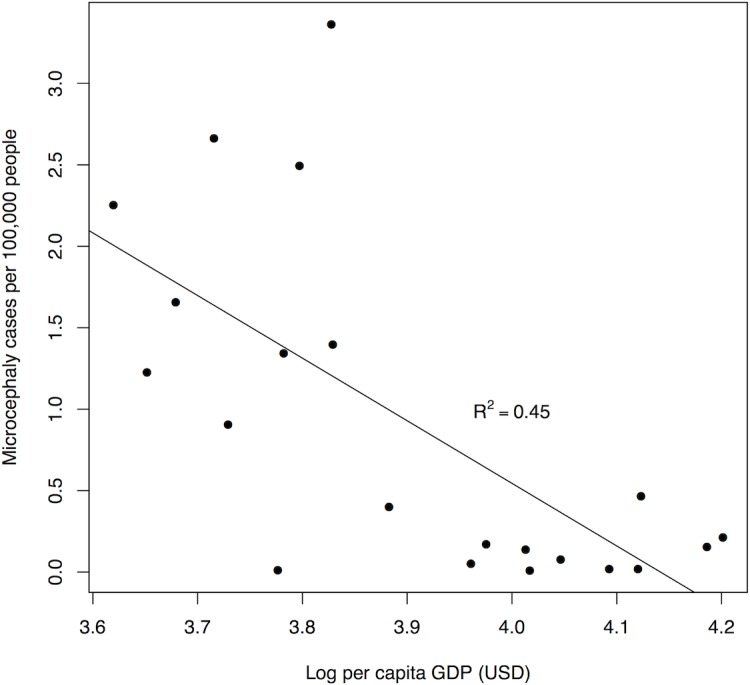
Confirmed ZIKV-linked microcephaly cases in Brazilian states versus per capita GDP (in log10 US Dollars). The number of confirmed ZIKV-linked microcephaly cases per 100,000 people (as of July 15, 2016) is negatively correlated (Pearson’s correlation coefficient r = -0.64) with per capita GDP for states with one or more cases. Total GDP data were retrieved from the 2012 IBGE report (http://www.ibge.gov.br/english/estatistica/economia/contasregionais/2012/default.shtm; accessed July 7, 2016). Population sizes by state were retrieved from the 2014 IBGE report. Per capita GDP = Total GDP/population size. Microcephaly data are from weekly epidemiological reports from PAHO, as compiled by the CDC EPI.

Poverty can create the ideal conditions for disease transmission. It affects access to running water, education, and health care. Together, these interacting symptoms of poverty lead to negative correlations between income and ZIKV risk. Across Brazilian states, higher per capita gross domestic product (GDP) is correlated with lower per capita rates of confirmed ZIKV-linked microcephaly cases, a proxy for ZIKV incidence (Fig 4). Periods of economic decline may force individuals into lower socioeconomic status, in which the symptoms of poverty promote ZIKV risk. In 2014, before ZIKV emerged in the Americas, Brazil fell into a recession and the unemployment rate jumped from 6.8% to 8.5% [57]. The ongoing recession may have increased the risk of ZIKV infection for a new group of vulnerable people, but this link requires further investigation.

### Human movement

Mobility between individuals of lower and higher socioeconomic status may extend ZIKV transmission risk to a wider demographic. On a national scale, human movement has predicted DENV epidemic dynamics in Pakistan [58]. At the local level, house-to-house movements based on social connections have shaped the geographic distribution of new cases of DENV in Iquitos, Peru [59], suggesting that routine travel from impoverished communities to wealthier neighborhoods may expand infection risk. In Brazil, slums are generally situated near metropolitan cities [55], so the close proximity of these socioeconomically distinct regions may also facilitate the spread of ZIKV across social boundaries.

*Ae*. *aegypti* and the arboviruses it spreads often follow human movement: historically, *Ae*. *aegypti*, YFV, and DENV spread globally with the slave trade and the shipping industry [15,16]. More recently, intercontinental air travel, which increases yearly, has expedited the spread of vector-borne diseases, especially in industrialized countries [60]. Long distance travel amplifies the international threat of ZIKV transmission by allowing pathogens including Ebola, SARS, and influenza to spread rapidly around the world [60–62]. Since air travel demand is expected to double by 2035 [63], it will become an even more effective means of spreading viruses globally. Particularly in asymptomatic cases, viruses can be easily transported globally with human hosts, supporting the hypothesis that ZIKV arrived in the Americas via human air travel.

### Social consequences of ZIKV emergence

Though ZIKV generally causes mild, self-limiting febrile illness [10], it has raised alarm for its link to GBS and microcephaly. Fetal ZIKV cases have been identified in pregnant women in 21 countries [64], and ZIKV-linked microcephaly has been confirmed in eight countries including Brazil (1,687 cases) and the US (15 cases) as of July 14, 2016 [6] (Fig 1). The coming years will see a cohort of children with unknown long-term health outcomes and caregivers with an immense social and economic burden. Fear of these rare conditions and sexually-transmitted ZIKV [65] will redefine public interest in the prevention and treatment of infectious diseases. As WHO Director-General Margaret Chan stated, “The response [to ZIKV] now requires a unique and integrated strategy that places support for women and girls of child-bearing age at its core” [66]. Women’s health has taken center stage in the public, the scientific community, and the press. The spread of information (and potentially misinformation) about the risks of ZIKV infection will likely affect women’s reproductive rights, tourism, and beyond.

### Perceived risk of disease

**Fig 5:**
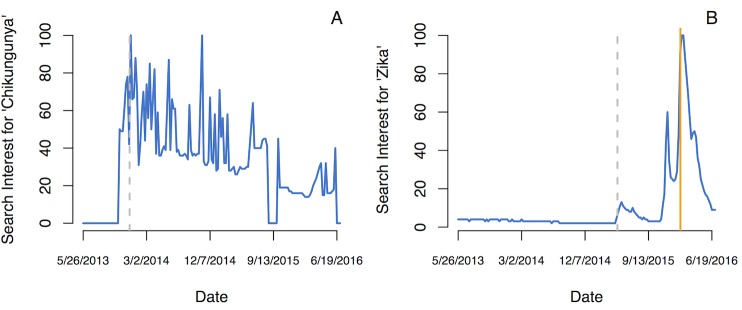
Search interest for “chikungunya” and “Zika.” Search interest, relative to the highest search volume between June 2013 and June 2016, for the terms “chikungunya” in St. Martin (A) and “Zika” in Brazil (B). A value of 100 represents the highest search volume recorded in each country within this time period, so direct comparison of relative search volume is possible without controlling for population or the number of Internet users. Gray dashed lines indicate the first cases of laboratory-confirmed CHIKV and ZIKV in the Americas. The orange bold line indicates declaration of a public health emergency of international concern (PHEIC) by WHO. Data are from Google Trends (https://www.google.com/trends/; accessed 5 July 2016).

Perceived risk of disease likely influences people’s behavior, beliefs, and health outcomes. In St. Martin and Brazil, where CHIKV and ZIKV emerged for the first time in the Americas, respectively, the onset of symptoms is a major sign of illness and thus a major indicator of disease risk [67, 68]. ZIKV, which is highly asymptomatic [8], does not comply with this perception of disease, while CHIKV, which shows symptoms in 75% of cases [69], does. We examined the influence of risk perception of CHIKV and ZIKV on public interest over time using Google Trends data for “Zika” searches in Brazil and “chikungunya” searches in St. Martin. CHIKV and ZIKV share similar transmission ecology and emerged in countries with similar perceptions of disease, but differ widely in their rate of symptomatic versus asymptomatic cases. As a result, we hypothesized that relative to the Google search volume in each country, searches for CHIKV in St. Martin would be consistently high, while searches for ZIKV in Brazil would be relatively low. CHIKV received more immediate attention than ZIKV, likely because it was initially perceived to pose a higher risk (Fig 5A). However, coincident with WHO’s declaration of a reported association between ZIKV and microcephaly (and a concomitant, sudden increase in perceived risk), searches for “Zika” drastically increased (Fig 5B). Beyond the impact of the perceived risk of ZIKV on public interest, the magnitude and intensity of public health responses to the ZIKV epidemic may also be linked to symptomatology and risk perception.

### Abortion and reproductive rights

The threat of microcephaly has expanded the cultural implications of ZIKV because women at risk of ZIKV infection may wish to postpone or terminate pregnancies. Outbreaks of viruses that cause birth defects are relatively rare, but an epidemic of rubella in the US in the 1960s caused congenital rubella syndrome (CRS) in the babies of infected mothers. This example may be illustrative as a model for social responses to teratogenic viruses during the current ZIKV epidemic. Though abortions were strictly regulated, in a study of rubella-infected pregnant women, most chose to terminate their pregnancy [70]. Using rubella as a model for ZIKV, we would expect that (potentially illicit) abortion rates, including miscarriages, in the Americas could increase above typical rates during the ZIKV epidemic; however, inadequate collection of abortion data in Latin America limits the scope of this hypothesis.

Safe abortions are not readily accessible for most women in Latin America. In Brazil, the United Nations has supported efforts to increase access to abortions, but some government officials have sought to restrict it further [71]. In a 2014 poll, 79% of Brazilians rejected the legalization of abortions [72]. Pope Francis has called abortion an “absolute evil” to be avoided in the interest of life, instead favoring contraception as “the lesser of two evils” [73, 74]. Amidst public, legislative, and religious backlash, the main government recommendation has been to postpone pregnancy [75]. The success of this response will likely be limited because unplanned pregnancy represented 56% of total pregnancies in Latin America in 2012 [76], likely due to limited access to contraceptives, lack of sex education, and high rates of sexual assault [77, 78]. These factors may also exacerbate the risk of sexually-transmitted ZIKV. Access to contraception and safe (legal or illegal) abortions will inevitably vary by socioeconomic status, potentially further exacerbating the differential impact of ZIKV on the urban poor. Because religious beliefs dictate most abortion policies, it is unclear if abortion policies will change in the wake of the ZIKV epidemic.

### Tourism and the 2016 summer olympics in Rio de Janeiro

Perceived risk of ZIKV infection poses a threat to tourism. For example, widespread public apprehension about ZIKV stirred doubts about the safety of the 2016 Summer Olympics in Rio de Janeiro. Historically, mass gatherings have been favorable for rapid transmission of infectious diseases [79]. As a result, some in the public health community and beyond suggested that the 2016 Summer Olympics should be moved or postponed. By contrast, Burattini et al. estimated that there is only a 3.5% chance of foreign tourists being bitten by an *Ae*. *aegypti* mosquito during the Summer Olympics, while the risk of becoming infected with ZIKV from a mosquito bite was estimated to be 1.8/1,000,000 [80]. Travel specifically for the Summer Olympics was projected to pose little additional risk of establishing autochthonous transmission by *Ae*. *aegypti* [81], and according to WHO, no athletes or visitors reported ZIKV infection during the Summer Olympics [82]. Nonetheless, tourism is likely to suffer from fears about ZIKV throughout the Americas. Travel warnings have extended to 59 countries and territories, including Latin America, Pacific Islands, Singapore, Cape Verde, and southern Florida. Perceived ZIKV risk, particularly for couples considering pregnancy, may impact tourism for the next several years.

## Conclusion

A suite of concomitant environmental and social changes facilitated global ZIKV emergence by altering the global ecology of vector transmission. In this intensified transmission regime, explosive epidemics of DENV, CHIKV, ZIKV, and other arboviruses are likely to continue to emerge. We showed direct correlations between ZIKV and poverty, deforestation, and natural disasters and circumstantial support for the role of warmer-than-average temperatures, declining vector control, unreliable sanitation access, recession, political corruption, and global travel. Together, these environmental and social changes affect mosquito distribution, habitat availability, human contact, and association with arboviruses, in turn promoting the spread of vector-borne diseases like ZIKV. Further experimental and observational work to measure the effects of environmental and social changes described here (Fig 2) on *Ae*. *aegypti* abundance and contact with humans, as well as epidemiological studies of risk factors for ZIKV exposure, susceptibility, and morbidity, are critical for responding to ZIKV and future arbovirus epidemics. This research will elucidate the multifactorial “perfect storm” of arbovirus emergence and resurgence, informing more proactive monitoring of existing and yet undiscovered vector-borne diseases.

**Key learning points**

- Deforestation is associated with elevated ZIKV risk. Less forest cover correlates with higher rates of confirmed ZIKV-linked microcephaly cases in Brazil.
- ZIKV transmission may be linked to poverty. Lower per capita GDP correlates strongly with higher rates of confirmed ZIKV-linked microcephaly cases in Brazil.
- Inadequate or damaged infrastructure may increase the abundance of mosquito breeding sites and thereby promote ZIKV transmission. Following a major earthquake in Manabi, Ecuador, the incidence of ZIKV cases in the province increased dramatically.
- In Brazil, capuchin monkeys and common marmosets may be important reservoirs for ZIKV. Both primates are physically and phylogenetically close to humans and have high rates of seroconversion for ZIKV.
- As a model for ZIKV, the rubella outbreak in the United States suggests that (potentially illicit) abortion rates may increase in the Americas as a result of the ZIKV epidemic.

**Top four papers**

1. Hales DS, de Wet N, Maindonald J, Woodward A. Potential effect of population and climate change on global distribution of dengue fever: an empirical model. The Lancet [Internet]. 2002 Sep 14;360(9336).
2. Hotez PJ. Neglected Tropical Diseases in the Anthropocene: The Cases of Zika, Ebola, and Other Infections. PLoS Negl Trop Dis. 2016 Apr 8;10(4):e0004648.
3. Stewart Ibarra AM, Ryan SJ, Beltrán E, Mejía R, Silva M, Muñoz Á. Dengue Vector Dynamics (Aedes aegypti) Influenced by Climate and Social Factors in Ecuador: Implications for Targeted Control. 2013 Nov;8(11).
4. Musso D, Cao-Lormeau VM, Gubler DJ. Zika virus: following the path of dengue and chikungunya? The Lancet. 2015 Jul;386(9990):243–4.

## Glossary

anthropogenic: originating from human activity
anthropophilic: preferentially feeding on humans
arboviruses: viral pathogens that are transmitted by infected arthropod species (also, *vector-borne diseases*)
autochthonous: locally-acquired, as compared with imported
confirmed case of ZIKV: patient who meets criteria for a *suspected case of ZIKV* and has laboratory confirmation of recent ZIKV infection. CDC defines laboratory confirmation is defined as having culture of ZIKV from blood, body fluid, or tissue; or detection of ZIKV antigen or viral ribonucleic acid (RNA) in serum, cerebrospinal fluid (CSF), placenta, umbilical cord, fetal tissue, or other specimen (e.g., amniotic fluid, urine, semen, saliva); or positive ZIKV immunoglobulin M (IgM) antibody test in serum or CSF with positive ZIKV neutralizing antibody titers and negative neutralizing antibody titers against dengue or other flaviviruses endemic to the region where exposure occurred.
confirmed case of ZIKV-linked microcephaly: live newborn who meets criteria for *suspected case of ZIKV-linked microcephaly* and ZIKV was detected in specimens of newborn
Guillain-Barré syndrome (GBS): an autoimmune disease that mostly causes short-term neurological complications including partial paralysis of the body
microcephaly: a neurological condition in which a baby is born with a skull size at least two standard deviations smaller than average
oviposit: to lay eggs
seroconversion: the development of specific, detectable antibodies in the blood following infection
source reduction: a vector control method that aims to prevent the development of mosquito larvae by eliminating potential breeding sites such as swamps and household water containers
suspected case of ZIKV: patient with rash and two or more of the following symptoms: fever, conjunctivitis, arthralgia, myalgia, or periarticular edema
suspected case of ZIKV-linked microcephaly: live newborn with microcephaly and whose mother traveled to/lived in area with ZIKV vectors during pregnancy or had unprotected sex with partner who traveled to/lived in area with ZIKV vectors. CDC defines definite microcephaly as head circumference (HC) at birth less than the 3rd percentile for gestational age and sex or if HC at birth is not available, HC less than the 3rd percentile for age and sex within the first 2 weeks of life.
sylvatic transmission cycle: transmission between wild animals and wildlife-feeding mosquitoes, which may occasionally spill over to infect humans
teratogen: an agent that causes birth defects when the mother is exposed during pregnancy
ultra-low volume space spraying: the aerial application of insecticides in the form of fine droplet particles that are used to control adult mosquito populations
urban transmission cycle: transmission in which anthropophilic mosquitoes transmit a pathogen from human to human, without requiring wildlife reservoirs
vector: an organism that transports a pathogen from one infected host to another
vector-borne diseases: infectious diseases that are transmitted by infected arthropod species including mosquitoes, ticks, and flies (also, *arboviruses*)
vectorial capacity: a measure of the transmission potential of a vector population, which describes the rate at which future infections arise from an infected host if the vectors are completely susceptible to the pathogen
vector competence: the ability of a vector to acquire, maintain, and transmit a pathogen
vector control: any intervention to suppress or replace vector populations that transmit pathogens
